# The Medical Profession in the United States

**Published:** 1900-09-08

**Authors:** 


### Hospital Clinics and Medical Progress.

### THE MEDICAL PROFESSION IN THE UNITED STATES.

THE address recently delivered by Dr. A. Jacobi, of New York, before the International Medical Congress, on the subject of Medicine and Physicians in the United States, contained much that was interesting as indicating the position now occupied by the medical profession in America. He said that there were now 156 medical schools in the United States, with 24,119 students, the number of the latter having increased 142 per cent. in the last 21 years. Of these 156 schools, 21 called themselves homœopathic, with 1,833 students; seven eclectic, with 852 students; three physio-medical, with 85 students. Seventy-four of the above-mentioned 156 schools were departments of colleges and universities, 82 were separate institutions, and 152 granted degrees. At present there were probably 120,000 practitioners in the United States, the ratio of physicians to population being less than 1 in 600, while in Great Britain it was 1 to 1,200, and in Russia 1 to 8,500. Proportionately to population the States had four times as many physicians as France, five times as many as Germany, six times as many as Italy, and six times as many medical schools as either of these countries. He proceeded to insist on the evils of having so many schools. Medical teaching would become better and more uniform, he thought, and more in accordance with the real requirements of the people, when these 156 medical schools had been reduced to 25, and when each of them should be connected with a University as its medical department. The power to confer degrees differed in the various schools of the 45 States composing the Union, and the low standards permitted in many of the professional schools was due to failure to subject the degree-giving power to strict State supervision. In New York and Pennsylvania the laws now prevented an abuse of the power of conferring degrees, but a Bill having the object of enforcing the same power in Illinois, although strongly advocated by educators, had been defeated by politicians in favour of low standards, while in Ohio and Nebraska the statutes required only the nominal endowment of 5,000 dollars for a degree-conferring institution. In other States and Territories any body of men might, as a rule, form an educational corporation with power to confer degrees, without any guarantee whatever that the privilege would not be abused; from all of which it was easy to see that it was impossssible to judge of the Union from a single State. Again, the power of every medical school to give a licence to practice together with its degree of doctor of medicine had led to the country being furnished with many low-grade practitioners. It was true that, mainly in the Eastern States, a few colleges were gradually improving their methods of teaching and lengthening their curricula, but they were exceptions to the rule. Even in New York some of the schools bitterly opposed every attempt at progress, and any such improvement as had taken place was due to the influence of the medical profession forcing the schools to improve their medical instruction. The great difficulty, in so great a country as the United States, was to obtain anything like a uniform standard among people so differently situated. Indeed, it might be said that every century and every people had exactly the doctors it deserved, just as it had the rulers it deserved, and thus it had been impossible to arrive at anything like a uniform standard all over the United States, or to arrange such reciprocity between them as to allow a doctor of one State to settle in another.

Turning from standards of education to standards of ethics, Dr. Jacobi said that in the United States it was considered unethical for a doctor to own a drug store or any part of it; ever to take a patent on any invention of his own; ever to recommend over his name a patented instrument, a proprietary food, or medicine, or mineral water. In fact, in most points the standard of ethics was that adopted in England, many of the rules being borrowed from a work published in England on the subject. In regard to the question of homœopathy, he said: "It is hardly necessary to say to those of you for whom the practice of medicine is not only diagnosis and autopsy, but the treatment and cure of the patient in whose behalf a consultation is to be held, that when medicinal treatment is in question you cannot agree with a homœopath who is a Hahnemanian; and you do not want to meet a homœopath who, because the name is still fashionable, and for a portion of the misinformed public the subject of an almost religious fanaticism, employs that title for meretricious purposes. Still there are cases in which it would be inhuman to refuse a consultation. Not only were such consultations held from olden times, but even in large cities exceptions to the rule have always been frequent, indeed too frequent in my opinion. Moreover, whoever is acquainted with smaller cities and villages, where a homœopath is the only rival or companion of the regular physician, knows that for either it would be suicidal to refuse a consultation. Only lately one of the medical men most widely known for his wisdom, in the American Medical Association and the profession at large, Dr. S. C. Busey, of Washington, proclaimed that the rule forbidding such consultations should be so modified or explained as to permit them in cases of emergency. This is exactly what the Medical Society of the State of New York made its official policy in 1882. We considered it more honest to admit by law what was constantly being done, and to decree precisely that for reasons of humanity and in an urgent case a consultation should not be refused. For this sin we, the body of the Medical Society of New York, were expelled from the American Medical Association."

Turning to the question of hospitals, Dr. Jacobi said that the hospitals and the operating-rooms in the United States were not only like those in Europe, but in many cases excelled them. There was no country in which the demands of antisepsis and asepsis were more scrupulously obeyed than in his. But he thought that the arrangements for medical attendance on hospitals was capable of improvement. In Europe a distinguished man was known for his life-long connection with a particular hospital. That was less so in the States. The ambition to be on a hospital staff, the democratic tendency of the authorities to be fair to the greatest possible number, were reasons why the hospital staffs were unduly large, why the year was divided up into alternating temporary services, and why men were compelled, in order to have hospital services throughout the year, to seek them in different institutions. The improprieties and drawbacks of this arrangement had, he said, been so long felt that the establishment of permanent services, most of them of a special character, had been resorted to in some hospitals. The assistantships were in almost every case in the hands of young graduates who obtained their places after competitive examinations, their terms of service ranging from one and a half to two and a half years, in semi-annual advances from grade to grade, a method which afforded a great many young physicians an opportunity for more or less independent work, guided by their superiors, who made their gratuitous daily visits during their term of office. All this was a great advantage, not only to them, but to the districts in which they afterwards practised.

[We may add that Dr. Jacobi's address does not seem to have been altogether pleasing to some of his fellow Americans, who hold that he was far too apologetic. —Ed. *T. H.*]

